# Association between life’s crucial 9 and lung health: a population-based study

**DOI:** 10.1186/s12890-025-03684-z

**Published:** 2025-05-03

**Authors:** Haolin Shi, Xiuhua Ma

**Affiliations:** Beijing Friendship Hospital, Beijing Daxing District People’s Hospital, Capital Medical University Daxing Teaching Hospital, Beijing, China

**Keywords:** Lung health, Life's crucial 9, Cardiovascular health, Machine learning, Shapley additive explanations

## Abstract

**Background:**

As a cardiovascular health (CVH) assessment tool, Life’s Crucial 9 (LC9) is often associated with diverse chronic health indicators. However, no study has yet explored the association of LC9 with multifactorial components of lung health. Thus, this study aimed to investigate the correlation of LC9 with lung health.

**Methods:**

This cross-sectional study used data from the National Health and Nutrition Examination Survey (NHANES), which covers individuals aged 40 years and older with complete LC9 and lung health data. Multiple regression was employed in linear relationships investigation, while Restricted Cubic Spline (RCS) was used to explore nonlinear relationships. Subgroup analyses and interaction tests demonstrated the stability of associations. Combining LC9 to build a Light Gradient Boosting Machine (LightGBM) machine learning (ML) model to predict lung health, Shapley Additive Explanations (SHAP) sorted the contribution of LC9 components to the model.

**Results:**

From a total of 10,461 study participants, 1725 had low CVH, 7476 had moderate CVH, and 1260 had high CVH. There was a strong positive correlation between LC9 score and lung health. This association remained consistent across subcomponent strata. RCS analysis revealed non-linear associations between LC9 and respiratory outcomes, including cough, asthma, and COPD. The LightGBM model incorporating LC9 demonstrated excellent predictive performance for lung health, with favorable metrics in Area Under the Curve (AUC), accuracy, and specificity. SHAP analysis identified depression, nicotine exposure, and BMI scores as the predominant contributors among LC9 components to the model’s predictive capability.

**Conclusion:**

Individuals with better CVH as assessed by LC9 tended to have better lung health. The combination of the LightGBM model could achieve better prediction results.

**Supplementary Information:**

The online version contains supplementary material available at 10.1186/s12890-025-03684-z.

## Background

Chronic respiratory diseases, including asthma and chronic obstructive pulmonary disease (COPD), are conditions that affect the airways and other structures of the lung [1, 2]. The primary risk factors for these diseases include biofuel toxicity, air pollution, and smoking [3]. Statistically, approximately 545 million people worldwide, accounting for 7.4% of the global population, are affected by chronic respiratory conditions [1]. Each year, 4 million people die from these diseases, making them the world’s third most common cause of death, following cardiovascular disease (CVD) and cancer [4]. With industrialization, urbanization, and population aging, the rising prevalence and mortality of chronic respiratory diseases are placing considerable strain on both human health and global healthcare systems. Cough, phlegm, and wheezing are common chronic respiratory symptoms that serve as key diagnostic and therapeutic indicators for a variety of chronic respiratory conditions. As such, it is crucial to investigate factors that are closely linked to lung health.

CVD is a global health challenge, alongside chronic respiratory diseases. Several studies have shown a close connection between both. For instance, 12% of patients with ischemic heart disease (IHD) also have comorbid COPD, which is also linked to poorer acute-phase outcomes and increased long-term mortality in IHD patients [5]. The mechanisms common to lung health and CVH are complex and involve the interaction of multiple factors. Some factors are non-modifiable, such as age and gender; others are modifiable, including glycemia, lipids, diet, sleep, and physical activity [6]. Developing prevention strategies for modifiable factors may even mitigate the adverse effects of non-modifiable factors. In order to advocate for the public to promote CVH by improving their lifestyles, the American Heart Association (AHA) introduced Life’s Essential 8 (LE8), which integrates multiple health behaviors (HB) and health factors (HF) in 2022 [7]. Previous studies have demonstrated that LE8 is positively associated with several respiratory symptoms, lung diseases, and lung function [6, 8]. In 2024, an article published in Circulation emphasized the role of mental health in CVH, suggesting that it be expanded to LC9 by adding LE8 [9]. Therefore, as a more comprehensive indicator for assessing CVH, the association between LC9 and lung health deserves further investigation.

ML has increasingly been applied in clinical predictive modeling. For example, the Machine Learning Mortality Prediction Model for COPD (MLMP-COPD) constructed by Matthew Moll et al. outperforms the remaining models in the COPDGene and ECLIPSE cohorts [10]. This study utilized NHANES data to determine whether LC9 and lung health are related, and to develop ML models aimed at elucidating the clinical predictive value of LC9-assessed lifestyle on lung health in the United States. This is the first investigation into the connection between LC9 and lung health, and applied a more innovative ML to build predictive models. It is hypothesized that LC9 may be positively correlated with lung health, and is better at predicting.

## Methods

### Study population

The National Center for Health Statistics authorized the NHANES survey, which collects data from a probability-based, multistage stratified sample of the country’s population for American health and nutritional status evaluation. All participants provided informed consent. The data from this study are publicly available in the NHANES database and allowed for further analysis by the public. The study complied with the Strengthening the Reporting of Observational Studies in Epidemiology (STROBE) reporting guideline [11].

The sample combined NHANES data from 2005 to 2012 and included participants aged 40 years or older with complete lung health and LC9 scores. Initially, 40,790 participants were included. After excluding participants with missing lung health (*N* = 26079) and LC9 score (*N* = 4250), a total of 11,061 participants entered the final analysis (see Additional file 1, Figure S1). Due to the lack of lung function data in NHANES 2005–2006, the relevant analyses only included NHANES 2007–2012 with complete forced expiratory volume in 1 s (FEV1) and forced vital capacity (FVC).

### Assessments of LC9 scores and lung health

The LC9 score includes assessments of four health behaviors (diet, physical activity, nicotine exposure, and sleep) and five health factors [BMI (body mass index), blood lipids, blood glucose, blood pressure, and mental health]. The study used the criteria from previously published NHANES-related articles to calculate the LC9 score, as described in the supplementary information (see Additional file 1, Table S1) [12]. Each CVH element received a score between 0 and 100. The aggregate LC9 score is the unweighted average of the nine CVH ratings. Based on the overall LC9 score, the CVH level was divided into three categories: low (0–49 points), medium (50–79 points), and high (80–100 points).

Lung health in this study was assessed by respiratory symptoms (cough, phlegm, wheeze), chronic lung disease (asthma, bronchitis, emphysema, COPD), and spirometry (FEV1, FVC, FEV1/FVC). Cough and phlegm were defined as having a cough or phlegm most of the time for at least three months of the year. A wheezing or whistling sound in the chest throughout the previous 12 months was considered wheezing. Participants were considered to have an associated chronic lung disease if a professional had told them that they had asthma, bronchitis, or emphysema. Spirometry (FEV1, FVC, FEV1/FVC) was measured using the Ohio 822/827 Dry Rolled Sealed Volumeter. The subjects took a deep breath in the standing position and then exhaled to the best of their ability. Subjects with a first measurement of FEV1/FVC < 0.7 were remeasured after inhalation of bronchodilators, and COPD was diagnosed if FEV1/FVC was still < 0.7.

### Covariate choice

The covariates included gender, age (year), race, education level, marital status, family income-to-poverty ratio (PIR), CVD, drinking, cancer or malignancy, fumes at work, second-hand smoke at home, family history of asthma, white blood cell (WBC) (103/uL), hemoglobin (Hb) (g/dL), platelet (PLT) (103/uL), eosinophil (103/uL), C-reactive protein (CRP) (mg/dL), 2-Naphthol (2-NAP) (ng/g), 1-Hydroxypyrene (1-PYR) (ng/g), lead (Pb) (ug/g), cadmium (Cd) (ug/g). CVD is defined as having been informed by a professional that you have suffered from any heart attack, stroke, congestive heart failure, coronary atherosclerosis, or angina pectoris. Drinking is defined as more than 12 drinks in a year. Measurement details of all covariates are available in the NHANES database.

### Statistical analyses

In this study, sample weights were considered for NHANES-based sampling (sampling weights: WTMEC4YR; stratification variable: SDMVSTRA; cluster identifier: SDMVPSU), and missing values of covariates were interpolated according to the Random Forest algorithm. This study first investigated differences in population characteristics stratified by CVH. Weighted means and standard deviations were calculated using the rank sum test to evaluate continuous variables, and weighted percentages were calculated using the chi-square test to evaluate categorical variables. The variance inflation factor (VIF) test did not screen for covariates with covariance (all VIF < 2). LightGBM screened covariates based on SHAP value ordering and built three models: unadjusted model 1; model 2 adjusted for covariates with the top 6 SHAP values; and model 3 adjusted for covariates with the top 13 SHAP values. Covariates included in each dependent variable were described in the supplementary information (see Additional file 1, Table S2). Because the OR/β value of LC9 in the regression analysis was too low to be observed, LC9 divided by 10 (LC9/10) was used instead of LC9. Subsequently, weighted multivariate regression in three models was employed to investigate the correlation between LC9 scores and lung health. RCS explores nonlinear associations, with the weighted median serving as a reference point. Next, associations within subgroups were discussed, and interaction tests were conducted to assess heterogeneity. Finally, the data were divided into an 80% training set and a 20% test set to build LightGBM prediction models for the seven categorical dependent variables when 30, 22, and 15 features were incorporated respectively. AUC, accuracy, and specificity were used to evaluate the performance of the model. SHAP analysis was used to rank the importance of the features. Statistical analyses were conducted in R 4.4.3 and Python 3.8.10 using Scikit-learn. Statistical significance was set at *P* < 0.05.

## Results

### Population baseline description

Of the 10,601 participants in this study, there were 1725 (13.48%) with low CVH, 7476 (70.25%) with moderate CVH, and 1260 (16.27%) with high CVH. The specific characteristics of the participants were described in detail according to CVH stratification (Table 1). Participants with higher CVH tended to be younger, female, Non-Hispanic White, better educated, with a partner, more affluent, without CVD, no fumes at work, no second-hand smoke at home, no family history of asthma, with lower WBC, Hb, PLT, eosinophil, CRP, 2-NAP, 1-PYR, Pb, and Cd. Participants with lower CVH were more likely to cough, phlegm, wheeze, and had a higher probability of developing asthma, emphysema, chronic bronchitis, COPD, with lower FEV1, FVC, FEV1/FVC compared to participants with higher CVH.

**Table 1:** Participants’ baseline traits categorized by CVH (weighted)

Characteristic	Cardiovascular health			
	Low	Moderate	High	*P*-value
N	1725 (13.48%)	7476 (70.25%)	1260 (16.27%)	
Age (year)	56.13 (55.45–56.81)	57.43 (56.89–57.97)	55.43 (54.64–56.22)	< 0.0001
Gender				< 0.0001
Male	809 (44.76%)	3903 (50.66%)	554 (40.50%)	
Female	916 (55.24%)	3573 (49.34%)	706 (59.50%)	
Race				< 0.0001
Mexican American	248 (6.27%)	1139 (5.87%)	122 (2.93%)	
Other Hispanic	133 (3.83%)	624 (3.69%)	75 (2.20%)	
Non-Hispanic White	791 (70.18%)	3836 (76.76%)	814 (85.35%)	
Non-Hispanic Black	501 (15.66%)	1509 (9.22%)	133 (3.81%)	
Other Race	52 (4.05%)	368 (4.46%)	116 (5.72%)	
Education level				< 0.0001
< High school	707 (30.69%)	2088 (17.67%)	122 (4.92%)	
High school	458 (31.77%)	1840 (25.65%)	178 (12.61%)	
> High school	560 (37.54%)	3548 (56.68%)	960 (82.47%)	
Marital status				< 0.0001
Married/living with partner	903 (56.28%)	4752 (68.53%)	928 (78.88%)	
Widowed/divorced/separated	666 (35.25%)	2178 (24.64%)	259 (16.27%)	
Never married	156 (8.47%)	546 (6.83%)	73 (4.85%)	
Drinking				0.0716
Yes	1218 (74.30%)	5212 (74.99%)	926 (78.64%)	
No	507 (25.70%)	2264 (25.01%)	334 (21.36%)	
Cardiovascular disease				< 0.0001
Yes	439 (22.00%)	1037 (11.31%)	98 (5.84%)	
No	1286 (78.00%)	6439 (88.69%)	1162 (94.16%)	
Cancer or malignancy				0.0793
Yes	179 (11.11%)	1002 (13.45%)	170 (12.58%)	
No	1546 (88.89%)	6474 (86.55%)	1090 (87.42%)	
Fumes at work				0.0040
Yes	93 (6.28%)	448 (7.15%)	45 (4.14%)	
No	1632 (93.72%)	7028 (92.85%)	1215 (95.86%)	
Second-hand smoke at home				< 0.0001
Yes	684 (41.34%)	1053 (14.09%)	27 (1.96%)	
No	1041 (58.66%)	6423 (85.91%)	1233 (98.04%)	
Family history of asthma				< 0.0001
Yes	408 (23.09%)	(16.73%)	(13.46%)	
No	1317 (76.91%)	6253 (83.27%)	1076 (86.54%)	
Family PIR	2.40 (2.24–2.56)	3.25 (3.16–3.34)	4.02 (3.91–4.14)	< 0.0001
WBC (10^3^/uL)	8.16 (8.01–8.31)	7.12 (7.04–7.19)	6.20 (6.09–6.31)	< 0.0001
Hb (g/dL)	14.41 (14.25–15.57)	14.30 (14.21–14.39)	14.08 (13.97–14.18)	< 0.0001
PLT (10^3^/uL)	269.62 (263.75-275.49)	254.18 (251.26–257.10)	240.74 (236.12-245.36)	< 0.0001
Eosinophil (10^3^/uL)	0.24 (0.23–0.25)	0.20 (0.20–0.21)	0.17 (0.16–0.18)	< 0.0001
CRP (mg/dL)	0.85 (0.78–0.92)	0.49 (0.47–0.51)	0.28 (0.24–0.31)	< 0.0001
2-NAP (ng/g)	1819.73 (1440.20-2199.26)	581.98 (361.45-802.52)	512.94 (259.79-766.09)	< 0.0001
1-PYR (ng/g)	3.14 (2.91,3.38)	1.99 (1.90, 2.07)	1.62 (1.46–1.78)	< 0.0001
Pb (ug/g)	0.0087 (0.0081–0.0093)	0.0077 (0.0075–0.0080)	0.0078 (0.0072–0.0083)	0.0278
Cd (ug/g)	0.0049 (0.0046–0.0053)	0.0038 (0.0037–0.0039)	0.0035 (0.0032–0.0038)	< 0.0001
Cough				< 0.0001
Yes	378 (23.44%)	758 (11.02%)	44 (2.90%)	
No	1347 (76.56%)	6718 (88.98%)	1216 (97.10%)	
Phlegm				< 0.0001
Yes	323 (19.07%)	685 (9.17%)	48 (3.28%)	
No	1402 (80.93%)	6791 (90.83%)	1212 (96.72%)	
Wheeze				< 0.0001
Yes	497 (30.32%)	968 (13.80%)	73 (6.58%)	
No	1228 (69.68%)	6508 (86.20%)	1187 (93.42%)	
Asthma				< 0.001
Yes	354 (20.88%)	837 (12.05%)	124 (11.28%)	
No	1371 (79.12%)	6639 (87.95%)	1136 (88.72%)	
Emphysema				< 0.001
Yes	115 (7.20%)	194 (2.33%)	7 (0.40%)	
No	1610 (92.80%)	7282 (97.67%)	1253 (99.60%)	
Chronic bronchitis				< 0.001
Yes	215 (13.05%)	453 (6.54%)	45 (3.80%)	
No	1510 (86.95%)	7023 (93.46%)	1215 (96.20%)	
COPD				< 0.001
Yes	72 (8.57%)	280 (7.77%)	23 (2.73%)	
No	878 (91.43%)	3855 (92.23%)	760 (97.27%)	
FEV1 (ml)	2660.62 (2586.82-2734.41)	2938.34 (2903.71-2972.98)	3120.92 (3058.21-3183.63)	< 0.001
FVC (ml)	3560.14 (3455.63-3664.65)	3902.27 (3857.10-3947.43)	4100.12 (4019.50-4180.74)	< 0.001
FEV1/FVC	0.75 (0.74–0.75)	0.75 (0.75–0.76)	0.76 (0.76–0.77)	0.0003
LC9 score	42.54 (42.18–42.89)	65.09 (64.74–65.44)	85.90 (85.54–86.25)	< 0.001

### Association between LC9 scores and lung health

Table 2 demonstrates the relationship between total LC9, HB, and HF scores with lung health (Table 2). In all three models, LC9 as continuous and categorical variables were significantly negatively associated with cough, wheeze, asthma, chronic bronchitis, emphysema, COPD, and positively associated with FEV1, FVC (all *P* < 0.05). The correlation remains in the vast majority of HB and HF scores. Only FEV1/FVC was not significantly associated with LC9 in Model 3, while it was significantly positively and negatively associated with HB and HF, respectively (*P* < 0.0001).

**Table 2:** Association between LC9 scores and respiratory symptoms (weighted)

	OR/β (95%CI), *P* value		
	Crude model (Model 1)	Minimally adjusted model (Model 2)	Fully adjusted model (Model 3)
Outcome: cough			
LC9 score/10	0.62 (0.59, 0.66), < 0.0001	0.63 (0.59, 0.67), < 0.0001	0.72 (0.67, 0.77), < 0.0001
Low	Reference	Reference	Reference
Moderate	0.40 (0.34, 0.48), < 0.0001	0.44 (0.36, 0.53), < 0.0001	0.61 (0.50, 0.76), < 0.0001
High	0.10 (0.07, 0.14), < 0.0001	0.11 (0.07, 0.15), < 0.0001	0.19 (0.13, 0.29), < 0.0001
*P* for trend	< 0.0001	< 0.0001	< 0.0001
Health behaviors score/10	0.72 (0.69, 0.75), < 0.0001	0.72 (0.69, 0.76), < 0.0001	0.80 (0.76, 0.85), < 0.0001
Health factors score/10	0.83 (0.79, 0.87), < 0.0001	0.82 (0.78, 0.86), < 0.0001	0.90 (0.85, 0.95), 0.0007
Outcome: phlegm			
LC9 score/10	0.65 (0.62, 0.70), < 0.0001	0.67 (0.63, 0.71), < 0.0001	0.71 (0.66, 0.76), < 0.0001
Low	Reference	Reference	Reference
Moderate	0.43 (0.35, 0.42), < 0.0001	0.47 (0.38, 0.58), < 0.0001	0.55 (0.44, 0.69), < 0.0001
High	0.14 (0.10, 0.21), < 0.0001	0.16 (0.11, 0.23), < 0.0001	0.23 (0.16, 0.34), < 0.0001
*P* for trend	< 0.0001	< 0.0001	< 0.0001
Health behaviors score/10	0.74 (0.71, 0.78), < 0.0001	0.75 (0.72, 0.79), < 0.0001	0.79 (0.75, 0.84), < 0.0001
Health factors score/10	0.84 (0.80, 0.88), < 0.0001	0.84 (0.80, 0.88), < 0.0001	0.91 (0.86, 0.96), 0.0017
Outcome: wheeze			
LC9 score/10	0.65 (0.62, 0.68), < 0.0001	0.68 (0.65, 0.72), < 0.0001	0.72 (0.68, 0.76), < 0.0001
Low	Reference	Reference	Reference
Moderate	0.37 (0.32, 0.43), < 0.0001	0.43 (0.37, 0.51), < 0.0001	0.52 (0.44, 0.61), < 0.0001
High	0.16 (0.12, 0.21), < 0.0001	0.21 (0.16, 0.28), < 0.0001	0.27 (0.20, 0.37), < 0.0001
*P* for trend	< 0.0001	< 0.0001	< 0.0001
Health behaviors score/10	0.78 (0.75, 0.80), < 0.0001	0.80 (0.78, 0.83), < 0.0001	0.85 (0.82, 0.89), < 0.0001
Health factors score/10	0.78 (0.75, 0.82), < 0.0001	0.81 (0.78, 0.85), < 0.0001	0.84 (0.80, 0.89), < 0.0001
Outcome: asthma			
LC9 score/10	0.81 (0.75, 0.87), < 0.0001	0.84 (0.78, 0.91), < 0.0001	0.83 (0.77, 0.89), < 0.0001
Low	Reference	Reference	Reference
Moderate	0.52 (0.43, 0.63), < 0.0001	0.60 (0.49, 0.74), < 0.0001	0.61 (0.49, 0.74), < 0.0001
High	0.48 (0.33, 0.70), 0.0003	0.61 (0.40, 0.91), 0.0200	0.58 (0.38, 0.87), 0.0123
*P* for trend	0.0004	0.0200	0.0123
Health behaviors score/10	0.90 (0.87, 0.94), < 0.0001	0.93 (0.89, 0.97), 0.0021	0.94 (0.90, 0.98), 0.0077
Health factors score/10	0.85 (0.80, 0.91), < 0.0001	0.87 (0.81, 0.93), 0.0002	0.87 (0.82, 0.94), 0.0003
Outcome: emphysema			
LC9 score/10	0.57 (0.53, 0.61), < 0.0001	0.59 (0.54, 0.65), < 0.0001	0.62 (0.55, 0.70), < 0.0001
Low	Reference	Reference	Reference
Moderate	0.31 (0.23, 0.40), < 0.0001	0.36 (0.25, 0.50), < 0.0001	0.41 (0.27, 0.61), 0.0001
High	0.05 (0.02, 0.13), < 0.0001	0.08 (0.03, 0.21), < 0.0001	0.10 (0.04, 0.27), < 0.0001
*P* for trend	< 0.0001	< 0.0001	< 0.0001
Health behaviors score/10	0.64 (0.61, 0.68), < 0.0001	0.63 (0.58, 0.68), < 0.0001	0.63 (0.58, 0.69), < 0.0001
Health factors score/10	0.83 (0.77, 0.90), < 0.0001	0.89 (0.81, 0.97), 0.0096	1.00 (0.90, 1.11), 0.9684
Outcome: chronic bronchitis			
LC9 score/10	0.71 (0.67, 0.76), < 0.0001	0.77 (0.71, 0.83), < 0.0001	0.77 (0.71, 0.82), < 0.0001
Low	Reference	Reference	Reference
Moderate	0.47 (0.38, 0.58), < 0.0001	0.58 (0.45, 0.74), 0.0001	0.61 (0.47, 0.78), 0.0004
High	0.26 (0.17, 0.41), < 0.0001	0.39 (0.24, 0.62), 0.0002	0.39 (0.24, 0.62), 0.0003
*P* for trend	< 0.0001	< 0.0001	0.0001
Health behaviors score/10	0.82 (0.79, 0.86), < 0.0001	0.86 (0.81, 0.90), < 0.0001	0.86 (0.81, 0.91), < 0.0001
Health factors score/10	0.82 (0.77, 0.88), < 0.0001	0.87 (0.82, 0.93), < 0.0001	0.90 (0.84, 0.96), 0.0020
Outcome: COPD			
LC9 score/10	0.85 (0.80, 0.91), < 0.0001	0.85 (0.79, 0.91), < 0.0001	0.85 (0.79, 0.91), < 0.0001
Low	Reference	Reference	Reference
Moderate	0.90 (0.65, 1.24), 0.5209	0.81 (0.58, 1.12), 0.2075	0.78 (0.57, 1.07), 0.1349
High	0.30 (0.20, 0.46), < 0.0001	0.28 (0.18, 0.44), < 0.0001	0.26 (0.17, 0.40), < 0.0001
*P* for trend	< 0.0001	< 0.0001	< 0.0001
Health behaviors score/10	0.83 (0.79, 0.88), < 0.0001	0.80 (0.75, 0.84), < 0.0001	0.77 (0.72, 0.82), < 0.0001
Health factors score/10	1.04 (0.97, 1.11), 0.3029	1.09 (1.02, 1.17), 0.0154	1.16 (1.08, 1.25), 0.0006
Outcome: FEV1			
LC9 score/10	100.13 (81.95, 118.31), < 0.0001	66.70 (52.97, 80.43), < 0.0001	53.02 (39.30, 66.75), < 0.0001
Low	Reference	Reference	Reference
Moderate	277.73 (187.98, 367.47), < 0.0001	139.87 (90.04, 189.71), < 0.0001	98.11 (45.25, 150.96), 0.0010
High	460.31 (362.71, 557.90), < 0.0001	268.62 (200.63, 336.62), < 0.0001	202.26 (131.10, 273.43), < 0.0001
*P* for trend	< 0.0001	< 0.0001	< 0.0001
Health behaviors score/10	35.49 (24.68, 46.29), < 0.0001	37.79 (28.24, 47.34), < 0.0001	25.62 (16.31, 34.93), < 0.0001
Health factors score/10	98.81 (81.08, 116.54), < 0.0001	38.46 (23.06, 53.86), < 0.0001	26.75 (10.86, 42.63), 0.0025
Outcome: FVC			
LC9 score/10	117.43 (93.45, 141.41), < 0.0001	78.41 (60.56, 96.26), < 0.0001	71.83 (54.04, 89.62), < 0.0001
Low	Reference	Reference	Reference
Moderate	342.13 (222.25, 462.00), < 0.0001	156.69 (86.18,227.20), 0.0001	139.83 (71.13, 208.53), 0.0004
High	539.98 (415.20, 664.76), < 0.0001	303.47 (219.68, 387.26), < 0.0001	267.40 (183.78, 351.01), < 0.0001
*P* for trend	< 0.0001	< 0.0001	< 0.0001
Health behaviors score/10	23.88 (9.42, 38.33), 0.0022	20.62 (8.63, 32.61), 0.0017	8.50 (-3.12, 20.13), 0.1619
Health factors score/10	142.77 (120.41, 165.13), < 0.0001	78.37 (61.57, 95.17), < 0.0001	71.98 (53.12, 90.83), < 0.0001
Outcome: FEV1/FVC			
LC9 score/10	0.0032 (0.0019, 0.0045), < 0.0001	0.0020 (0.0006, 0.0034), 0.0069	0.0009 (-0.0005, 0.0022), 0.2232
Low	Reference	Reference	Reference
Moderate	0.0069 (0.0003, 0.0135), 0.0447	0.0070 (0.0004, 0.0137), 0.0444	0.0027 (-0.0040, 0.0093), 0.4360
High	0.0152 (0.0081, 0.0223), 0.0001	0.0111(0.0034, 0.0188), 0.0074	0.0056 (-0.0021, 0.0133) < 0.1657
*P* for trend	0.0001	0.0075	0.1518
Health behaviors score/10	0.0048 (0.0038, 0.0058), < 0.0001	0.0057 (0.0047, 0.0067), < 0.0001	0.0056 (0.0046, 0.0066), < 0.0001
Health factors score/10	-0.0024 (-0.0040, -0.0058),0.0046	-0.0058 (-0.0075, -0.0041),<0.0001	-0.0068 (-0.0086, -0.0051), < 0.0001

Health behaviors score/10 and health factors score/10 in Model 3 added adjustments to each other

**Fig. 1:**
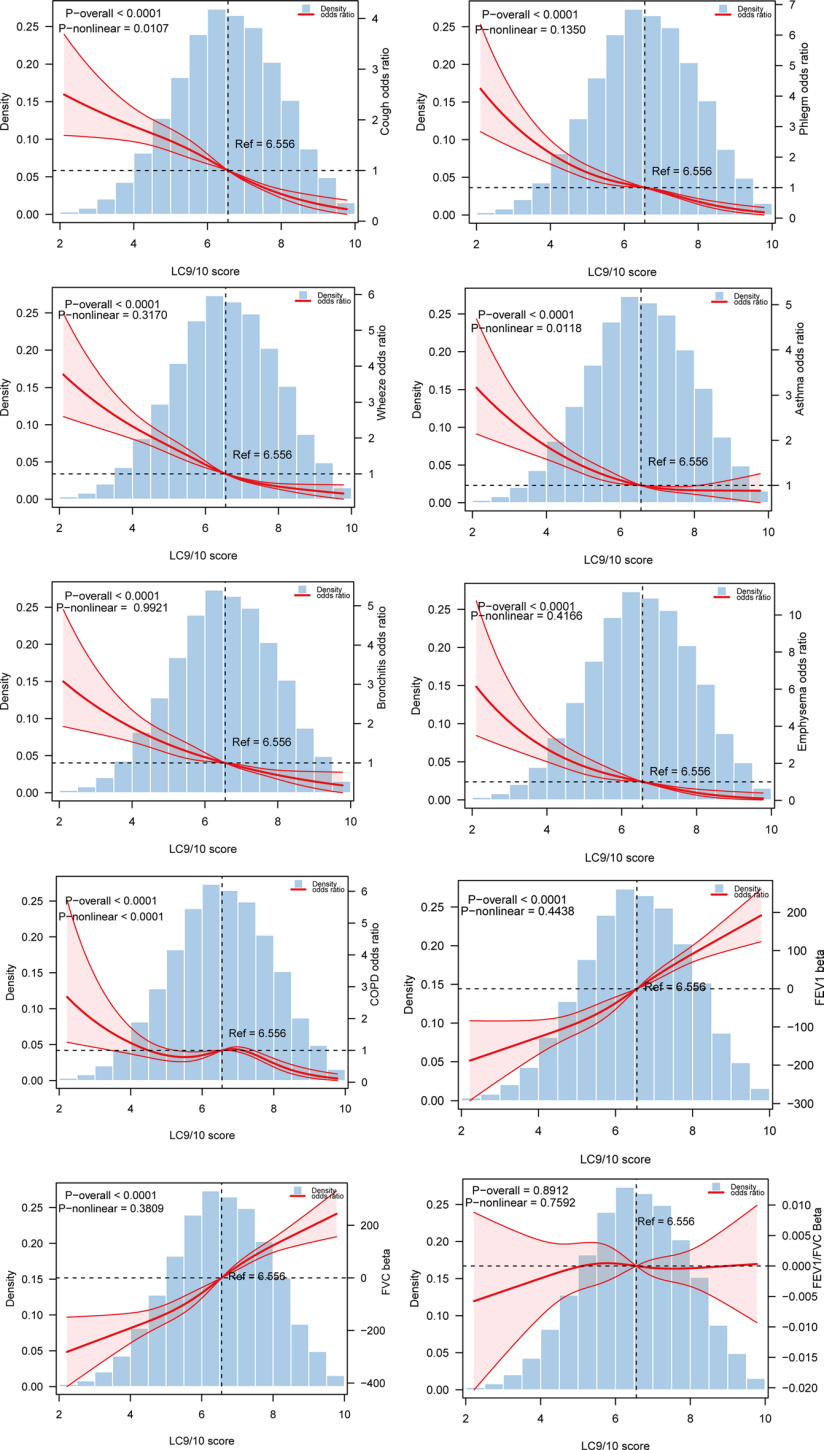
Restricted Cubic Spline analysis between LC9 and lung health (weighted) Model 3 was applied

RCS analysis showed a nonlinear relationship between LC9 and cough, asthma, and COPD (*P*-nonlinear < 0.05) (Fig. 1). The curves for cough and asthma fell steeply and then smoothed out, suggesting that the benefit of raising LC9 was greater at lower scores and that the effect stabilized or diminished with further increases in the score. The curve for COPD had an inverted U-shape, with the association being positive at lower LC9 and negative at higher scores. This suggests that there is a benefit in reducing COPD by raising LC9 to a sufficiently high level.

### Subgroup analyses

**Fig. 2:**
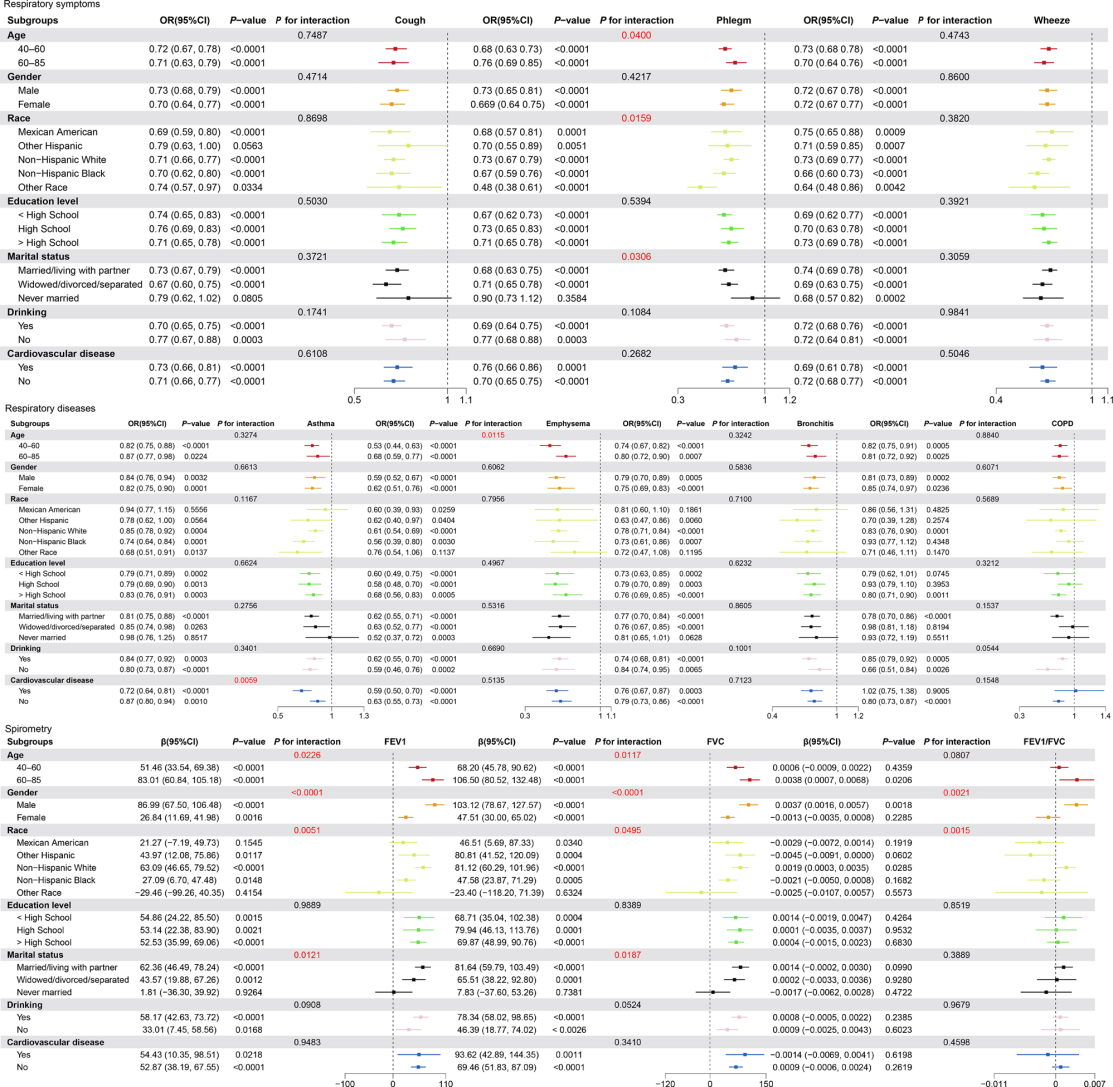
Analysis of the relationship between LC9 score and lung health by subgroup (weighted) Model 3 was applied, and *P* for interaction < 0.05 was labeled red

Further subgroup analyses suggested that The correlation between LC9 and lung health remained stable in the majority of strata (*P < 0*.05), and was significantly different between a few subgroups (*P* for interaction < 0.05) (Fig. 2).

### Toward a machine learning model for predicting lung health

Based on SHAP values assessed by LightGBM, the study selected the top 6, 13, 21 covariates and LC9 components (total top 15, 22, 30 features), applied them to LightGBM algorithms to generate the lung health risk predictive model (Table 3). Cough, asthma, COPD reached the highest AUC at the top 15 features; bronchitis at the top 22 features; phlegm, wheeze, emphysema at the top 30 features. The best AUC was emphysema at the top 30 features, reaching 0.889. All models had accuracy and specificity greater than 0.7.

**Table 3:** Metrics of LightGBM model in predicting lung health

Outcome	AUC	Accuracy	Specificity
Cough			
Top 30 variables	0.733	0.784	0.819
Top 22 variables	0.743	0.795	0.830
Top 15 variables	0.753	0.774	0.804
Phlegm			
Top 30 variables	0.727	0.783	0.814
Top 22 variables	0.716	0.776	0.809
Top 15 variables	0.705	0.771	0.799
Wheeze			
Top 30 variables	0.716	0.738	0.778
Top 22 variables	0.715	0.740	0.781
Top 15 variables	0.713	0.735	0.773
Asthma			
Top 30 variables	0.680	0.763	0.815
Top 22 variables	0.676	0.758	0.809
Top 15 variables	0.681	0.737	0.777
Bronchitis			
Top 30 variables	0.693	0.825	0.863
Top 22 variables	0.695	0.820	0.858
Top 15 variables	0.660	0.782	0.814
Emphysema			
Top 30 variables	0.889	0.919	0.935
Top 22 variables	0.886	0.913	0.929
Top 15 variables	0.865	0.892	0.904
COPD			
Top 30 variables	0.769	0.866	0.907
Top 22 variables	0.765	0.865	0.903
Top 15 variables	0.771	0.847	0.875

**Fig. 3:**
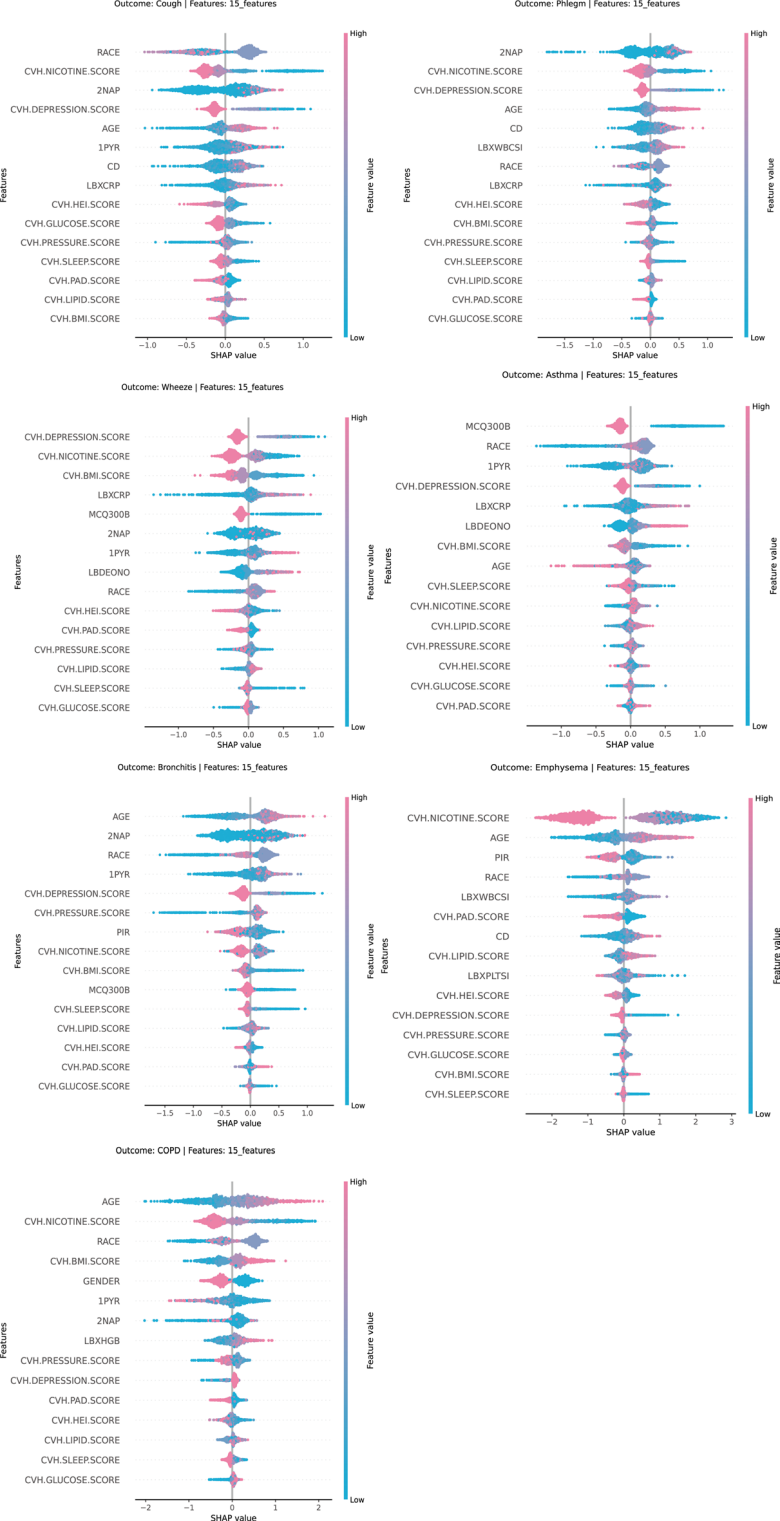
SHAP values of LC9 components for lung health prediction as determined by LightGBM LBXCRP: CRP, LBXWBCSI: WBC, LBXPLTSI: PLT, LBXHGB: Hb, LBDEONO: eosinophil, MCQ300B: family history of asthma

SHAP analysis was conducted to rank the importance of LC9 components in lung health prediction (Fig. 3). Nicotine and depression scores in LC9 appeared most frequently among the top five contributing characteristics to each outcome, with five occurrences each, followed by BMI scores, which appeared twice. Except for the high BMI score, which contributed positively to COPD, the remaining high scores contributed negatively to the outcome.

## Discussion

To ascertain the relationship between LC9 score and lung health, this study examined a large amount of NHANES demographic data. These results demonstrated a significant association between LC9 score and lung health (cough, wheeze, asthma, chronic bronchitis, emphysema, COPD, FEV1, FVC). RCS analysis showed a nonlinear relationship between LC9 and cough, asthma, COPD. The independence and stability of this association were confirmed through subgroup analysis and interaction tests. The LightGBM model was established to predict lung health; AUC, accuracy, and specificity showed good performance. SHAP analysis showed a greater contribution of depression, nicotine, and BMI scores among the LC9 components to predict lung health. The results suggest that the LC9 score can be used to assess lung health and that managing the HB and HF reflected by the LC9 score may help reduce the incidence of various respiratory symptoms and chronic lung diseases.

LC9 established by LE8 incorporating mental factors, prior research has indicated several long-term respiratory conditions association of LE8 and mental health. Data from the UK Biobank (2006–2010) revealed that the likelihood of developing chronic non-communicable respiratory diseases decreased by 20% for every 10-point increase in LE8 [13]. Numerous studies in both the US and the UK have also demonstrated a negative correlation between LE8 and asthma as well as COPD [13–15]. For the Singapore Longitudinal Ageing Study (SLAS) and NHANES, depression was positively associated with the progression of lung obstruction and COPD [16, 17]. Historically, research on lung health has focused on individual lifestyle factors, many of which are components of LC9. Among health behaviors, a healthy diet—such as the Dietary Approaches to Stop Hypertension and increased intake of polyunsaturated fatty acids [18, 19], moderate exercise (e.g., Liuzijue and Baduanjin exercises) [20, 21], smoking cessation, and reductions in blood nicotine levels [22], as well as healthy sleep patterns [23, 24], have been shown to improve respiratory symptoms, enhance lung function, and reduce the risk of chronic respiratory diseases. Regarding health factors, high BMI, blood glucose, blood lipids, blood pressure, and depression are all recognized as risk factors for lung injury. For example, functional residual capacity and expiratory reserve volume decrease exponentially with increasing BMI [25]. A medical examination survey in China indicated that lung function begins to decline at pre-diabetic stages, with more severe deterioration at higher blood glucose levels [26]. NHANES III showed a positive correlation between FEV1 with high-density lipoprotein and a negative correlation with low-density lipoprotein (LDL) [27]. A German survey showed that hypertension is associated with reduced FEV1 and forced vital capacity [28]. A NHANES study showed that depression was positively associated with COPD, with BMI playing a moderating role, and that the effect of depression on COPD risk was lower in the low BMI group than in the normal BMI group A NHANES study showed that depression was positively associated with COPD, with BMI playing a moderating role, and that the effect of depression on COPD risk was lower in the low BMI group than in the normal BMI group [17]. These factors tend to interact rather than act in isolation, and LC9 incorporates all of them, potentially exerting a more significant influence on lung health. This is consistent with this study’s conclusion that people with higher LC9 scores tend to have better lung health.

The potential mechanisms linking LC9 to lung health can be inferred from its components. Dietary fiber produces circulating short-chain fatty acids under fermentation by the gut microbiota, which attenuates inflammatory responses in the lungs through activation of free fatty acid receptors and epigenetic regulation [29, 30]. Saturated fat, trans fat, and cholesterol diets are major airway pro-inflammatory factors, while n-3 and n-6 fatty acids have anti-inflammatory properties [31]. Regular exercise benefits the airway’s smooth muscle layers and breathing patterns [32], and improved fitness is associated with a higher threshold for dyspnea [33]. Smoking cessation has been shown to reverse pathological and inflammatory changes in the lungs, including reducing airway epithelial cell proliferation, limiting airflow obstruction, and gradually normalizing inflammatory biomarkers [22]. Short sleep duration, on the other hand, may hyperactivate the airways by affecting eosinophil activation pathways, thereby increasing the risk of central obesity and elevated leptin levels, both of which impair lung function [23]. Leptin/leptin receptor signaling can promote obesity-associated asthma by inducing M1 macrophage polarization [34], leading to nonallergic asthma characterized by excessive neutrophil infiltration in the airways. Inflammation in this context is driven by circulating free fatty acids and hypertriglyceridemia in macrophages and other immune cells [35]. Lipotoxicity is further exacerbated by intracellular cholesterol accumulation and lipoprotein signaling, particularly via LDL [36, 37]. High blood sugar impairs immune function and increases the virulence of infectious microorganisms, thus heightening the risk of lung infections [38]. Additionally, hyperinsulinemia and insulin resistance can induce bronchial hyperresponsiveness through alterations in parasympathetic signaling [39]. β2-adrenergic blockers used in hypertension management may trigger bronchospasm, reduce lung function, and slightly decrease respiratory muscle strength [28]. Depression contributes to lung tissue damage through oxidative stress, reduces immune function through hypothalamic-pituitary-adrenal (HPA) axis hyperactivation, and affects the inflammatory response in the lungs through neuroendocrine disorders such as abnormal cortisol levels. Depressed people typically exhibit low self-efficacy and poor coping skills in the face of illness, which may affect their ability to self-manage their lung health [17]. These factors are interconnected and work synergistically within the respiratory system. For example, high-fat diets can increase both BMI and lipid levels [40], while smoking and high blood sugar can damage blood vessel walls, contributing to hypertension [41, 42]. Depressed patients often coexist with poor lifestyles such as smoking, physical inactivity, and unhealthy diets, and have lower adherence to treatment, thus exacerbating the progression of lung disease [17]. Of concern, high BMI has two sides to lung health: on the one hand, obesity is associated with chronic inflammation and metabolic disorders, increasing the burden on the respiratory system; on the other hand, the Obesity Paradox shows that in some COPD patients, mortality is lower in obese patients, which may be due to higher nutritional reserves and greater anti-inflammatory capacity [17]. The LC9 score combines these various factors, making it a comprehensive indicator that can more effectively assess lung health.

In addition, the dose-response relationship in this study showed that the benefit of LC9 boosting was greater for cough and asthma at lower CVH and for COPD at higher CVH. Subgroup analyses showed that the lung health benefits of boosting LC9 were greater in specific populations, such as cough in younger, other race, partnered individuals; asthma in CVD patients; emphysema in younger individuals; and lung function in older, male, non-Hispanic White, partnered individuals. It follows that attention needs to be paid to the effects of age, gender, race, marital status, and CVD on this association, and studies of LC9 in different demographic characteristics are warranted in the future.

Compared with traditional statistical methods, ML models can handle high-dimensional data and complex nonlinear relationships, making them more effective at capturing intricate data patterns and achieving higher predictive accuracy in clinical prediction. In this study, the LightGBM model combined with LC9 performed excellently in predicting chronic respiratory diseases. LightGBM is an efficient, distributed, and high-accuracy ML algorithm based on the gradient boosting framework. It uses an innovative histogram-based decision tree algorithm that significantly improves training speed and memory efficiency while maintaining high accuracy. In an NHANES study on dietary antioxidants and cardiovascular-cancer comorbidity, LightGBM achieved an AUC of 0.951, outperforming other ML models [43]. SHAP analysis showed that among the components of LC9, depression, nicotine, and BMI scores were relatively important for predicting lung health. Some other components, although significantly associated with lung health in traditional regression analysis, contributed less in the prediction model, suggesting that certain risk factors may lack strong predictive value for the disease.

This study has several strengths. The sample was drawn from a multistage and complex national survey, making the findings broadly applicable to the U.S. population. The inclusion of confounders adds confidence to the statistical results, and the large sample size allows for meaningful subgroup analysis. A more advanced ML was used to build the predictive model. However, there are certain limitations. First, as a cross-sectional study, causality cannot be determined. Second, self-reported data on lung health may introduce recall bias, and subjectivity reduces the precision of lung injury indications. Third, unmeasured or unaccounted confounders could have influenced the results. Fourth, a large number of participants were excluded because of missing values for lung health or LC9 calculations, possibly introducing selection bias. Finally, this study was confronted with only specific countries and ethnicities, limiting generalizability to other populations. Thus, further exploration of the relationship between CVH and lung health in diverse populations is warranted.

## Conclusion

There is a positive correlation between LC9 score and lung health. The LightGBM model combined with LC9 was excellent for its prediction. Clinicians should consider using LC9 as a guiding framework to manage patients’ lifestyles and improve both cardiovascular and respiratory health.

## Electronic supplementary material

Below is the link to the electronic supplementary material.

Supplementary Material 1

## Data Availability

The datasets generated during and analysed during the current study are available in the [NHANES] repository, [www.cdc.gov/nchs/nhanes/]. The database is open to the public.

## Abbreviations

CVH: Cardiovascular health
LC9: Life’s Crucial 9
NHANES: National Health and Nutrition Examination Survey
RCS: Restricted Cubic Spline
LightGBM: Light Gradient Boosting Machine
ML: Machine learning
SHAP: Shapley Additive Explanations
AUC: Area Under the Curve
COPD: Chronic obstructive pulmonary disease
CVD: Cardiovascular disease
IHD: Ischemic heart disease
AHA: American Heart Association
LE8: Life’s Essential 8
HB: Health behaviors
HF: Health factors
MLMP-COPD: Machine Learning Mortality Prediction Model for COPD
STROBE: Strengthening the Reporting of Observational Studies in Epidemiology
BMI: Body mass index
FEV1: Forced expiratory volume in 1 s
FVC: Forced vital capacity
PIR: Income-to-poverty ratio
WBC: White blood cell
Hb: Hemoglobin
PLT: Platelet
CRP: C-reactive protein
2-NAP: 2-Naphthol
1-PYR: 1-Hydroxypyrene
Pb: Lead
Cd: Cadmium
VIF: Variance inflation factor
SLAS: Singapore Longitudinal Ageing Study
LDL: Low-density lipoprotein
HPA: Hypothalamic-pituitary-adrenal
