# Supplementary material for: Association between life’s crucial 9 and lung health: a population-based study
Source: BMC Pulm Med. 2025 May 3;25:213. doi: 10.1186/s12890-025-03684-z (PMC12048992; doi:10.1186/s12890-025-03684-z)
Supplement: Supplementary file 1 — Supplementary Material 1 [file 12890_2025_3684_MOESM1_ESM.pdf]

Article title: Association between Life's Crucial 9 and lung health: a population-based study

Journal name: BMC Pulmonary Medicine

Haolin Shi [1], Xiuhua Ma [1*]

1. Beijing Daxing District People’s Hospital, Beijing Friendship Hospital, Capital Medical University

* Corresponding author

Corresponding author: Xiuhua Ma

Address: No.26 Huangcun West Street, Daxing District, Beijing, PR China

Postal code: 102699

Tell: 19979016239

Email: dxqrmyy@126.com

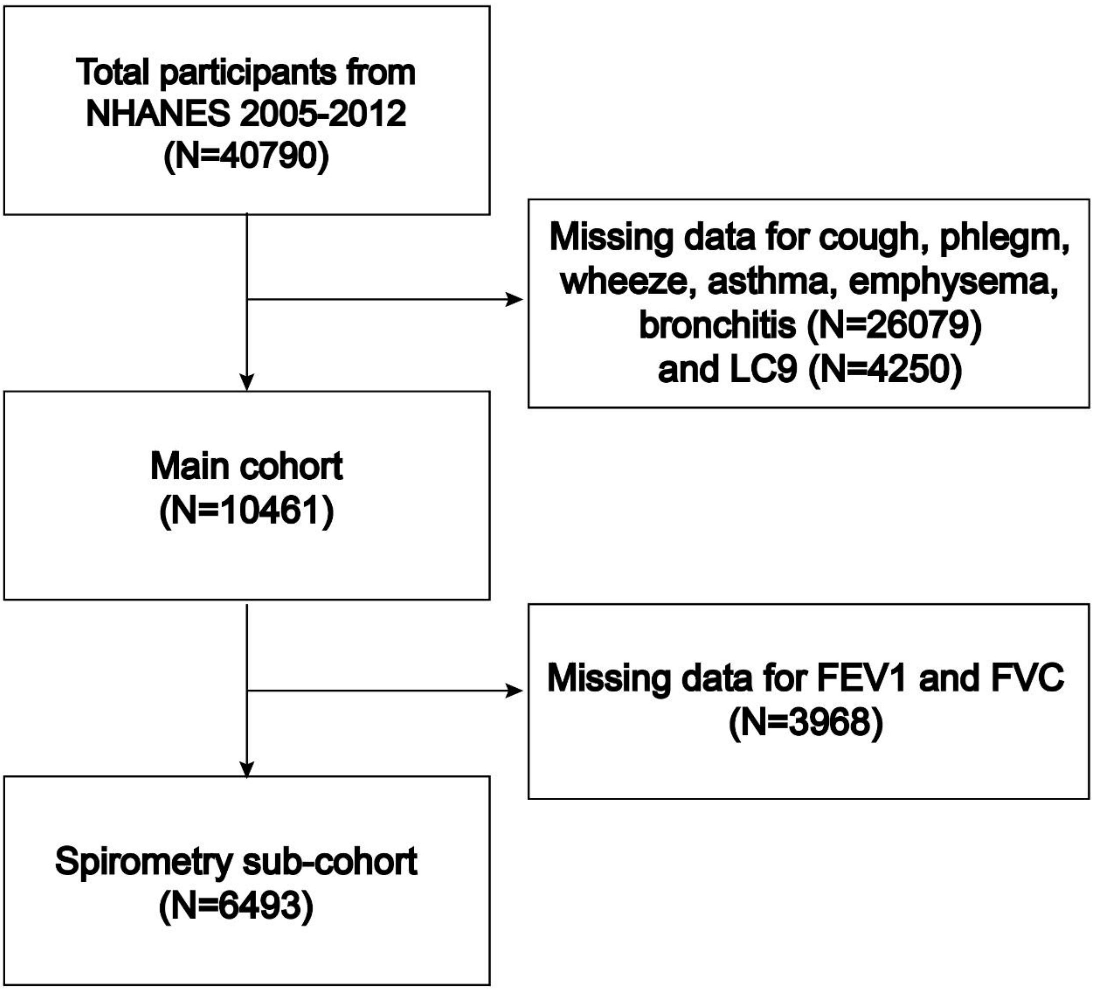

Figure S1. Flowchart of the NHANES 2005–2012 sample selection process

Table S1. Definition and scoring approach for the Life's Crucial 9 score

Domain	CVH Metric	Measurement	Quantification and Scoring of CVH Metric
Psychological Health	Depressive symptoms	Depression Screener Questionnaire (DPQ)	**Metric:** Nine-item depression screening instrument PHQ-9. Each symptom item in PHQ-9 is scored on a 4-point scale, from 0 ('not at all') to 3 ('nearly every day'), resulting in a total score of 0 to 27 points.<br><br>**Scoring:**<br>Points Level<br>100 the score of 0 to 4 points<br>70 the score of 5 to 9 points<br>30 the score of 10 to 14 points<br>0 the score of 15 to 27 points
Health	Diet	Healthy	Quantiles of DASH-style diet adherence

Behaviors		Eating Index-2015 diet score percentile	**Scoring (Population):**<br><u>Points</u> <u>Quantile</u><br>100 ≥95th percentile (top/ideal diet)<br>80 75th – 94th percentile<br>50 50th – 74th percentile<br>25 25th – 49th percentile<br>0 1st – 24th percentile (bottom/least ideal quartile)
	Physical activity	Self-reported minutes of moderate or vigorous physical activity per week	**Metric:** Minutes of moderate (or greater) intensity activity per week<br><br>**Scoring:**<br><u>Points</u> <u>Minutes</u><br>100 ≥150<br>90 120 – 149<br>80 90 – 119<br>60 60 – 89<br>40 30 – 59<br>20 1 – 29<br>0 0
	Nicotine exposure	Self-reported use of cigarettes or inhaled nicotine-delivery system	**Metric:** Combustible tobacco use and inhaled NDS use; or secondhand smoke exposure<br><br>**Scoring:**<br><u>Points</u> <u>Status</u><br>100 Never smoker<br>75 Former smoker, quit ≥5 yrs<br>50 Former smoker, quit 1 - <5 yrs<br>25 Former smoker, quit <1 year, or currently using inhaled NDS<br>0 Current smoker<br><br>Subtract 20 points (unless the score is 0) for living with an active indoor smoker in the home
	Sleep health	Self-reported average hours of sleep per night	**Metric:** Average hours of sleep per night<br><br>**Scoring:**<br><u>Points</u> <u>Level</u><br>100 7 – <9<br>90 9 – <10<br>70 6 – <7<br>40 5 – <6 or ≥10

			20 4 – <5<br>0 <4
Health Factors	Body mass index	Body weight (kg) divided by height squared (m^2)	**Metric:** Body mass index (kg/m^2)<br><br>**Scoring:**<br><u>Points Level</u><br><br>100 <25.0<br>70 25.0 – 29.9<br>30 30.0 – 34.9<br>15 35.0 – 39.9<br>0 ≥40.0
	Blood lipids	Plasma total and HDL-cholesterol with the calculation of non-HDL-cholesterol	**Metric:** Non-HDL-cholesterol (mg/dL)<br><br>**Scoring:**<br><u>Points Level</u><br>100 <130<br>60 130 – 159<br>40 160 – 189<br>20 190 – 219<br>0 ≥220<br><br>If the drug-treated level, subtract 20 points
	Blood glucose	Fasting blood glucose or casual hemoglobin A1c	**Metric:** Fasting blood glucose (mg/dL) or Hemoglobin A1c (%)<br><br>**Scoring:**<br><u>Points Level</u><br>100 No history of diabetes and FBG <100 (or HbA1c < 5.7)<br>60 No diabetes and FBG 100 – 125 (or HbA1c 5.7-6.4) (Pre-diabetes)<br>40 Diabetes with HbA1c <7.0<br>30 Diabetes with HbA1c 7.0 – 7.9<br>20 Diabetes with HbA1c 8.0 – 8.9<br>10 Diabetes with Hb A1c 9.0 – 9.9<br>0 Diabetes with HbA1c ≥10.0
	Blood pressure	Appropriately measured systolic and diastolic blood pressure	**Metric:** Systolic and diastolic blood pressure (mm Hg)<br><br>**Scoring:**<br><u>Points Level</u><br>100 <120/<80 (Optimal)<br>75 120-129/<80 (Elevated)

			50 130-139 or 80-89 (Stage I HTN)<br>25 140-159 or 90-99<br>0 ≥160 or ≥100<br><br>Subtract 20 points if treated level

Table S2. Three model covariates based on SHAP value ranking

	Model-adjusted covariates		
**Outcome**	**Crude model (Model 1)**	**Minimally adjusted model (Model 2)**	**Fully adjusted model (Model 3)**
Cough	no covariates	2-NAP, race, Cd, 1-PYR, CRP, age	2-NAP, race, Cd, 1-PYR, CRP, age, WBC, CVD, PLT, Hb, second-hand smoke at home, Pb, eosinophil
Phlegm	no covariates	2-NAP, Cd, age, WBC, race, CRP	2-NAP, Cd, age, WBC, race, CRP, 1-PYR, eosinophil, Hb, PIR, CVD, PLT, gender
Wheeze	no covariates	family history of asthma, CRP, 2-NAP, 1-PYR, eosinophil, race	family history of asthma, CRP, 2-NAP, 1-PYR, eosinophil, race, Cd, Pb, PIR, Hb, WBC, age, CVD
Asthma	no covariates	family history of asthma, race, 1-PYR, eosinophil, CRP, age	family history of asthma, race, 1-PYR, eosinophil, CRP, age, Hb, WBC, 2-NAP, pb, cd, gender, PLT
Emphysema	no covariates	age, PIR, race, WBC, Cd, PLT	age, PIR, race, WBC, Cd, PLT, 2-NAP, CVD, CRP, Hb, 1-PYR, Pb, family history of asthma
Bronchitis	no covariates	2-NAP, race, age, 1-PYR, PIR, family history of asthma	2-NAP, race, age, 1-PYR, PIR, family history of asthma, Hb, gender, WBC, education level, CRP, Pb, Cd
COPD	no covariates	age, gender, race, 2-NAP, 1-PYR, Hb	age, gender, race, 2-NAP, 1-PYR, Hb, WBC, PIR, PLT, Pb, Cd, eosinophil, CRP
FEV1	no covariates	age, race, education level, gender, CRP, PIR	age, race, education level, gender, CRP, PIR, cd, WBC, CVD, PLT, drinking, eosinophil, 1-PYR
FVC	no covariates	age, race, education level, gender, CRP, PIR	age, race, education level, gender, CRP, PIR, drinking, CVD, PLT, WBC, cd, Hb, 2-NAP
FEV1/FVC	no covariates	age, race, eosinophil, gender, WBC, Hb	age, race, eosinophil, gender, WBC, Hb, CRP, drinking, 2-NAP, PIR, cd, family history of asthma, PLT
